# Supplementary material for: Evaluating the Feasibility of a Low-Field Nuclear Magnetic Resonance (NMR) Sensor for Manure Nutrient Prediction
Source: Sensors (Basel). 2022 Mar 22;22(7):2438. doi: 10.3390/s22072438 (PMC9002543; doi:10.3390/s22072438)
Supplement: Supplementary file 1 [file sensors-22-02438-s001.zip › sensors-1615338-supplementary.pdf]

sensors

MDPI

Supplemental Material

Article

Evaluating the Feasibility of a Low-Field Nuclear Magnetic Resonance (NMR) Sensor for Manure Nutrient Prediction

Xiaoyu Feng *, Rebecca A. Larson and Matthew F. Digman

Department of Biological Systems Engineering, University of Wisconsin-Madison, 460 Henry Mall, Madison, WI 53706, USA; rebecca.larson@wisc.edu (R.A.L.); digman@wisc.edu (M.F.D.)
* Correspondence: xfeng43@wisc.edu; Tel.: +1-864-986-2404

Citation: Feng, X.; Larson, R.A.; Digman, M.F. Evaluating the Feasibility of a Low-Field Nuclear Magnetic Resonance (NMR) Sensor for Manure Nutrient Prediction. *Sensors* **2022**, *22*, 2438. https://doi.org/10.3390/s22072438

Academic Editor: Rebecca Re

Received: 12 February 2022
Accepted: 19 March 2022
Published: 22 March 2022

Publisher's Note: MDPI stays neutral with regard to jurisdictional claims in published maps and institutional affiliations.

Copyright: © 2022 by the authors. Submitted for possible open access publication under the terms and conditions of the Creative Commons Attribution (CC BY) license (https://creativecommons.org/licenses/by/4.0/).

Table S1. The precision of licensed parameters of NMR sensor provided by the manufacturer.

Parameter	RTs (min)	NMR SD (mg L^{-1})
TN	15	140
	30	99
	45	81
	60	70
NH_4-N	15	140
	30	99
	45	81
	60	70
TP	30	64
	45	52
	60	45
	90	37

NMR SD = standard deviation of NMR corresponding to 1 hr measurement time.

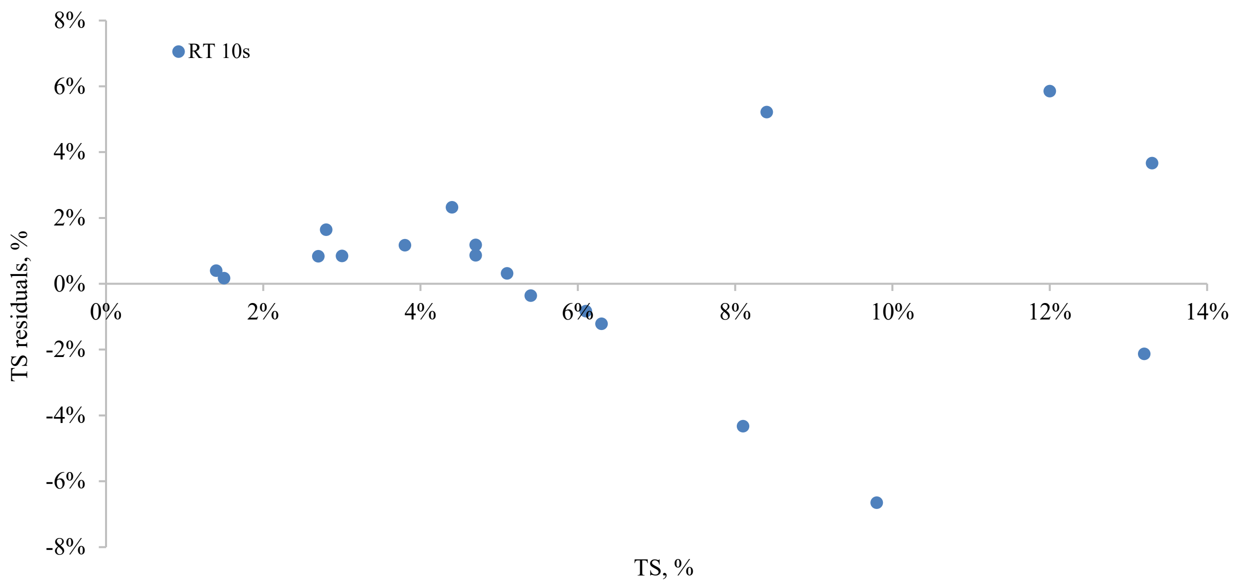

Figure S1. Residuals of TS between NMR predicted and lab measured versus the actual TS of samples (lab measurement) at 10s RT.

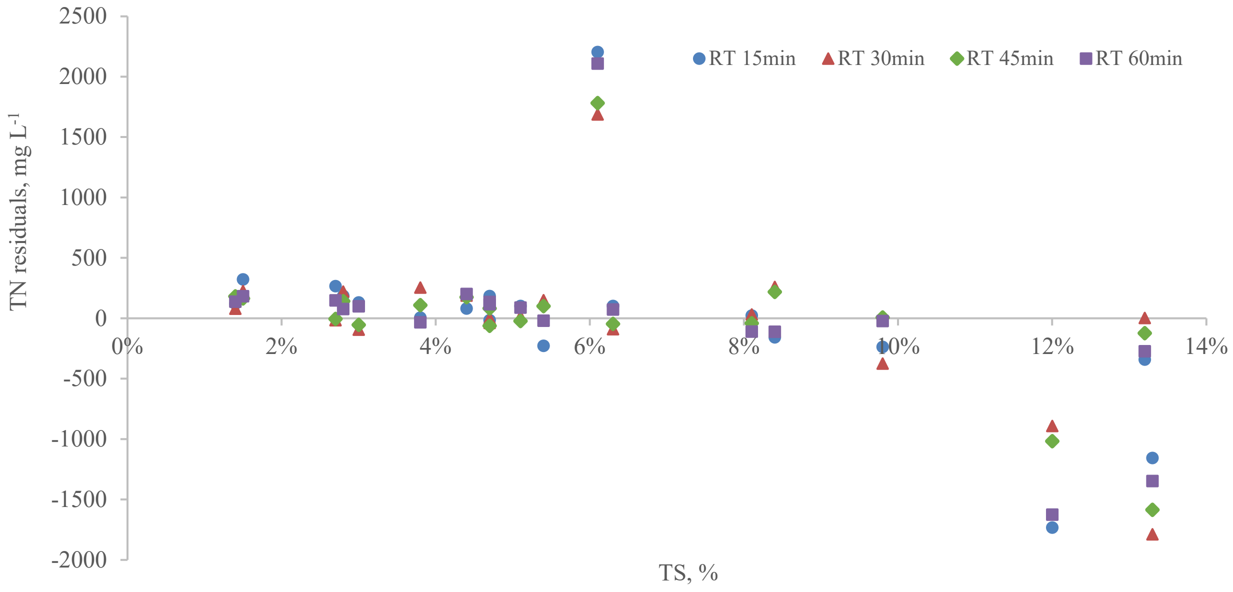

Figure S2. Residuals of TN between NMR predicted and lab measured versus the actual TS of samples (lab measurement) at 15 min, 30 min, 45 min, and 60 min RTs. .

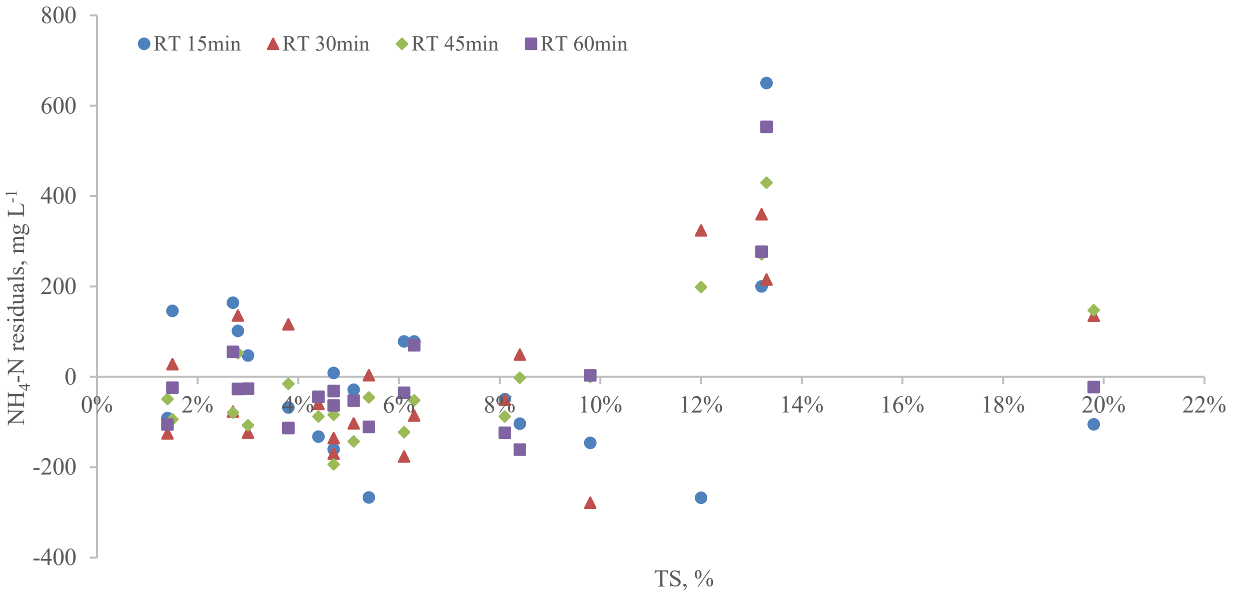

Figure S3. Residuals of NH_4-N between NMR predicted and lab measured versus the actual TS of samples (lab measurement) at 15 min, 30 min, 45 min, and 60 min RTs.

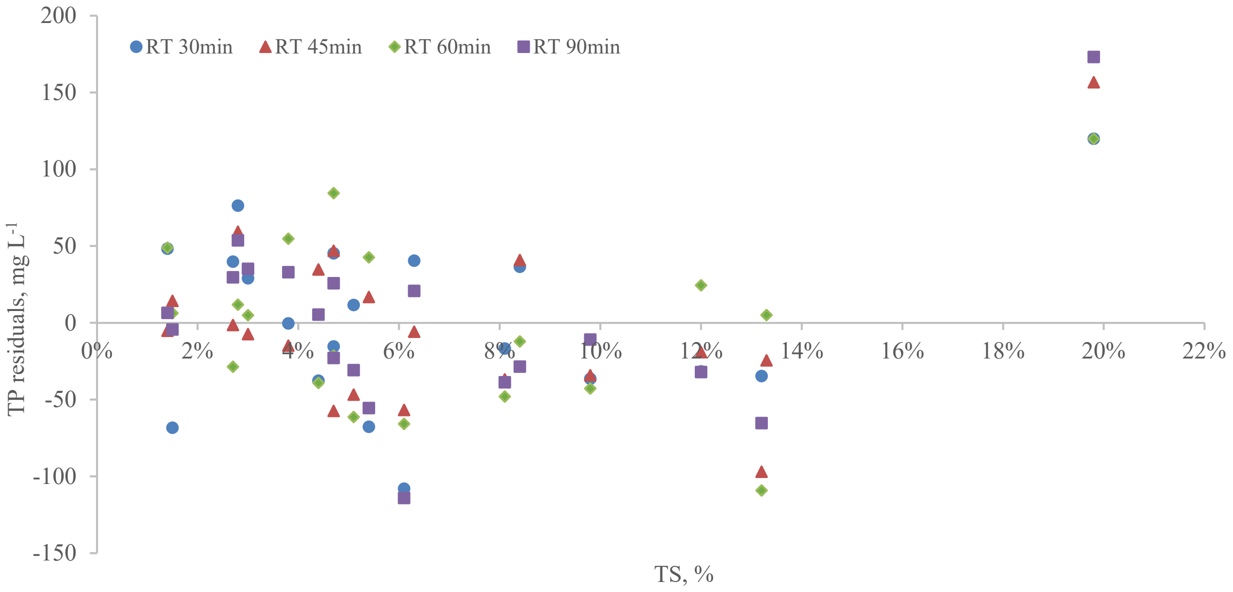

Figure S4. Residuals of TP between NMR predicted and lab measured versus the actual TS of samples (lab measurement) at 30 min, 45 min, 60 min, and 90 min RTs.
